# Contribution of the PhoP/Q regulon to survival and replication of *Salmonella enterica* serovar Typhimurium in macrophages

**DOI:** 10.1099/mic.0.048926-0

**Published:** 2011-07

**Authors:** Jessica A. Thompson, Mei Liu, Sophie Helaine, David W. Holden

**Affiliations:** Section of Microbiology, Centre for Molecular Microbiology and Infection, Imperial College London, Armstrong Road, London SW7 2AZ, UK

## Abstract

The ability of serovars of *Salmonella enterica* to cause systemic disease is dependent upon their survival and replication within macrophages. To do this, bacteria must withstand or surmount bacteriostatic and bactericidal responses by the host cell, including the delivery of hydrolytic enzymes from lysosomes to the phagosome. The bacterial two-component regulatory system PhoP/Q has been implicated in avoidance of phagolysosomal fusion by *S. enterica* serovar Typhimurium (*S.* Typhimurium) in murine macrophages. In this study, the involvement of PhoP/Q-activated genes in avoidance of phagolysosomal fusion was analysed: of all the *S.* Typhimurium mutant strains tested, only an *mgtC* mutant strain partially reproduced the phenotype of the *phoP* mutant strain. As this gene is required for bacterial growth in magnesium-depleted conditions *in vitro*, the contributions of PhoP/Q to intramacrophage replication and survival were reappraised. Although PhoP/Q was required for both replication and survival of *S.* Typhimurium within murine macrophages, subsequent analysis of the kinetics of phagolysosomal fusion, taking account of differences in the replication rates of wild-type and *phoP* mutant strains, provided no evidence for a PhoP/Q-dependent role in this process. PhoP/Q appeared to act subsequent to the process of phagolysosomal avoidance and to promote replication of those bacteria that had already escaped a phagolysosomal fate. Therefore, we conclude that the PhoP/Q regulon enables *S*. Typhimurium to adapt to intramacrophage stresses other than phagolysosomal fusion.

## INTRODUCTION

*Salmonella enterica* serovar Typhi (*S.* Typhi) causes typhoid fever, and is restricted to infection of primates. By contrast, *S. enterica* serovar Typhimurium (*S.* Typhimurium) has a broad host range and causes both self-limiting gastroenteritis and systemic diseases, depending upon the host; the systemic disease that it causes in susceptible mouse strains is frequently used as a model system to study typhoid fever. The ability of both serovars to cause systemic disease depends on their capacity to survive and grow within cells of the granulocyte/monocyte lineage, such as macrophages. Accordingly, *S.* Typhi is adapted to grow in human macrophages, while *S.* Typhimurium grows preferentially in mouse macrophages (Schwan *et al.*, 2000). Two multi-functional virulence systems contribute to the intramacrophage growth and virulence of *S. enterica*: the PhoP/Q two-component system, which activates the expression of many genes following bacterial uptake into phagosomes (Groisman *et al.*, 1989; Miller *et al.*, 1989), and the SPI-2 type III secretion system (T3SS) (Hensel *et al.*, 1995; Ochman & Groisman, 1996; Shea *et al.*, 1999), which translocates numerous effector proteins across the phagosomal membrane.

Macrophages use several bactericidal and bacteriostatic processes that must be counteracted, avoided or withstood for intracellular *S. enterica* to survive and replicate. DNA-, protein- and membrane-damaging reactive oxygen species are produced in the first few minutes of phagocytosis by NADPH oxidase activity. Toxic reactive nitrogen species are produced later, by inducible NO synthase (iNOS). The increased growth of *S.* Typhimurium in macrophages and knockout mouse strains lacking NADPH oxidase or iNOS demonstrates that bacteria are susceptible to these responses (Mastroeni *et al.*, 2000; Vazquez-Torres *et al.*, 2000a, b). This sensitivity is limited by the activity of bacterial detoxifying enzymes and regulatory factors, including catalases, hydroperoxidases (Hébrard *et al.*, 2009), superoxide dismutases (Craig & Slauch, 2009; De Groote *et al.*, 1997), HmpA, NorV, NrfA (Mills *et al.*, 2008) and σ^E^ (Testerman *et al.*, 2002).

A major process by which macrophages kill phagocytosed bacteria is the fusion of lysosomes to phagosomes to form phagolysosomes, acidic compartments containing proteases, glycosidases and lipases (Garin *et al.*, 2001). Numerous studies have shown that *S.* Typhimurium modifies its phagosome, known as the *Salmonella*-containing vacuole (SCV), such that it acquires some features of late endosomes but remains distinct from normal phagolysosomes. Interactions with early endosomes, characterized by recruitment of early endosomal markers (Hashim *et al.*, 2000), and acidification of the SCV (Drecktrah *et al.*, 2006; Rathman *et al.*, 1996, 1997) are followed by the acquisition of late endosome-associated proteins, including lysosomal membrane glycoproteins such as LAMP-1 (Becken *et al.*, 2010; Rathman *et al.*, 1997). However, SCVs are relatively deficient in mannose-6-phosphate receptors (M6PRs), which deliver hydrolytic enzymes to late endosomal/lysosomal compartments, and their cargo, such as cathepsin L, is also largely absent (Hashim *et al.*, 2000; Rathman *et al.*, 1997). Mature SCVs have also been found to be relatively inaccessible to lysosomes, as judged by co-localization with endocytotic lysosomal content using electron (Buchmeier & Heffron, 1991) and light (Becken *et al.*, 2010; Garvis *et al.*, 2001) microscopy. These observations were recently confirmed using an *in vitro* system that analysed fusion events between purified early or mature SCVs and lysosomes. In both cases, the majority of SCVs containing viable bacteria avoided fusion (Becken *et al.*, 2010).

Although the mechanism for avoidance of phagolysosomal fusion by *S.* Typhimurium in macrophages is not well characterized, both the SPI-2 T3SS (Uchiya *et al.*, 1999) and the PhoP/Q regulon (Garvis *et al.*, 2001) have been implicated. Our laboratory has reported that by 16 h after bacterial uptake by macrophages, an increased percentage of *phoP* mutant bacteria co-localizes with lysosomal markers compared with wild-type bacteria (Garvis *et al.*, 2001). In this study we analysed genes of the PhoP/Q regulon with respect to avoidance of phagolysosomal fusion by intramacrophage *S.* Typhimurium, and re-examined the method used to measure this process. A reappraisal of the role of PhoP/Q in bacterial replication and survival leads us to conclude that the PhoP/Q regulon is not directly involved in the avoidance of phagolysosomal fusion, but is required to promote the intramacrophage replication of *S.* Typhimurium.

## METHODS

### 

#### Bacterial strains and growth conditions.

The bacterial strains used in this study are listed in Supplementary Table S1. Bacteria were routinely grown in Luria–Bertani (LB) broth (Sambrook & Russell, 2001) at 37 °C, 200 r.p.m., except where otherwise indicated. Antibiotics were used at the following concentrations: kanamycin, 25 µg.ml^−1^; carbenicillin, 50 µg.ml^−1^; chloramphenicol, 50 µg.ml^−1^. Protein expression was induced with 0.2 % (w/v) l-arabinose, as required (Loessner *et al.*, 2007).

#### Bacterial mutagenesis.

*S.* Typhimurium LT2 mutant strains were constructed using the one-step λ red recombinase chromosomal inactivation method (Datsenko & Wanner, 2000). Primer sequences used for targeted mutagenesis and verification of recombination are listed in Supplementary Tables S2 and S3, respectively. Clean deletions of *phoP* and *pmrA* in *S.* Typhimurium LT2 were generated using FLP recombinase expressed from pCP20 to excise antibiotic-resistance cassettes.

#### Plasmids.

Plasmids used in this study are listed in Supplementary Table S1. pKD3 and pKD4 from *Escherichia coli* BW25141 (Datsenko & Wanner, 2000) were used as PCR templates for bacterial mutagenesis. pKD46 from BW25113, and pCP20 from BT340 (Datsenko & Wanner, 2000) were used to electroporate *S.* Typhimurium LT2 strains for the generation of mutant strains and the removal of antibiotic-resistance cassettes, respectively (Datsenko & Wanner, 2000). pDiGc (Helaine *et al.*, 2010) was electroporated into *S.* Typhimurium 12023 wild-type and Δ*phoP* : : *kan* strains (Helaine *et al.*, 2010). The vector pDSRED.T3_S4T (Sörensen *et al.*, 2003) was used to construct pBAD*mgtC* and pBAD*phoP*: *mgtC* and *phoP* were amplified from the *S.* Typhimurium genome with primers mgtCNdeI-F and mgtCHindIII-R, or phoPXbaI-F and phoPHindIII-R (Supplementary Table S2), which introduced a *Nde*I, *Hin*dIII or *Xba*I restriction site flanking the genes. Vector and PCR products were restriction-digested, and inserts were ligated into the vector in place of the excised *dsred.T3_S4T* gene under the regulation of the arabinose-inducible P_BAD_ promoter.

#### Identification of lysosomal compartments.

RAW264.7 (91962702) macrophage-like cells purchased from the European Collection of Cell Cultures were seeded on glass coverslips in 24-well plates at a density of 1×10^5^ cells per well. Cells were incubated for 12–24 h in Dulbecco's modified Eagle's medium (DMEM) supplemented with 10 % (v/v) fetal calf serum (FCS) at 37 °C, 5 % CO_2_, prior to experiments. To label lysosomes, macrophages were pulsed for 30 min with DMEM/10 % FCS containing 50 µg ml^−1^ Texas red ovalbumin (TROva; Molecular Probes, Invitrogen), washed, and incubated for 2 h in label-free DMEM/10 % FCS (Garvis *et al.*, 2001) before fixation with 3 % (w/v) paraformaldehyde (PFA) for 15 min at 25 °C. Cells were permeabilized with 0.1 % (w/v) saponin in PBS and labelled with a 1 : 500 dilution of rabbit anti-cathepsin D (anti-CtsD) primary antibody (provided by S. Kornfeld, Washington University) and a 1 : 200 dilution of anti-rabbit Cy2-conjugated secondary antibody (all conjugated secondary antibodies were purchased from Jackson Immunoresearch Laboratories). Co-localization between CtsD and TROva was analysed in a minimum of 50 cells per coverslip by laser-scanning confocal microscopy (Zeiss Axiovert LSM510).

#### Assay for SCV–lysosome interactions in macrophages.

RAW264.7 macrophages were seeded, and lysosomes were labelled with TROva as described above. Cells were infected at an m.o.i. of 10 : 1 with bacteria grown to stationary phase and opsonized as previously described (Beuzón *et al.*, 2000). A positive control containing heat-killed bacteria (500 µl stationary phase wild-type *S*. Typhimurium culture, incubated at 65 °C for 25 min) was similarly opsonized and used for infection. The lack of viability of these bacteria was confirmed by an absence of detectable c.f.u. on LB agar (Sambrook & Russell, 2001). At the time points indicated, infected macrophages were fixed with PFA, permeabilized with 0.1 % saponin in PBS, and labelled with a 1 : 200 dilution of goat anti-*Salmonella* CSA-1 primary (Kirkegaard and Perry Laboratories) and 1 : 200 anti-goat Cy2-conjugated secondary antibodies. Co-localization of heat-killed bacteria and CtsD was detected in macrophages labelled with anti-CtsD antibody and a 1 : 400 dilution of anti-rabbit Rhodamine Red-X (RRX)-conjugated secondary antibodies. Pixel-to-pixel co-localization between bacteria and lysosomal markers was assessed by laser-scanning confocal microscopy in a minimum of 50 cells per coverslip. Macrophages containing in excess of approximately 25 bacteria were excluded from the analysis due to the difficulty in accurately determining their number and the proportion co-localizing with TROva.

#### Bacterial growth in RAW264.7 macrophages.

RAW264.7 macrophages were infected for different time periods with opsonized stationary-phase cultures of bacteria at an m.o.i. of 10 : 1, as described above. Cells were lysed with 0.1 % (v/v) Triton X-100 in PBS, and c.f.u. were enumerated by plating serial dilutions of the lysate on LB agar. Bacterial growth was measured as the fold change in c.f.u. ml^−1^ recovered from macrophages between two time points.

#### Bacterial replication and survival in macrophages.

Bone marrow-derived macrophages (BMM) were extracted from BALB/c mice (Charles River) and cultured as described by Helaine *et al.* (2010). BMM medium [RPMI 1640 (Invitrogen) supplemented with 10 % (v/v) FCS Gold (PAA laboratories), 2 mM glutamine, 50 µM β-mercaptoethanol, 1 mM sodium pyruvate and 10 mM HEPES] was used to maintain BMM, which were seeded at a density of 2×10^5^ cells per well in 24-well plates. RAW264.7 macrophages or BMM were infected with bacterial strains carrying the pDiGc plasmid grown to late stationary phase in MgM-MES pH 5.0 [170 mM MES, pH 5.0, 5 mM KCl, 7.5 mM (NH_4_)_2_SO_4_, 0.5 mM K_2_SO_4_, 1 mM KH_2_PO_4_, 10 mM MgCl_2_, 38 mM glycerol and 0.1 % Casamino acids (Beuzón *et al.*, 1999)], in the presence of 0.2 % l-arabinose and antibiotics as required. At specified time points, cells were washed and lysed, and bacterial growth was quantified by enumeration of c.f.u. ml^−1^. The remaining lysate was centrifuged at 10 000 ***g***, bacteria were resuspended in 500 µl PBS, and the levels of DsRED and GFP fluorescence intensity were analysed by flow cytometry. Replication was measured as the fold change in the geometric mean of DsRED fluorescence intensity between two time points. Killing indices were calculated as the difference between replication rate and net growth rate in number of generations per hour, using the relationship *F* = 2*^n^*, where *n* corresponds to the number of generations and *F* is the fold change between the two time points.

#### Flow cytometric acquisition and analysis.

Bacterial samples were analysed using a FACSCalibur (Beckton Dickinson) for fluorescence intensities in the FL-1 (GFP) and FL-2 (DsRED) channels; a minimum of 10 000 bacterial events were analysed for each sample. Data were analysed using FlowJo 8.6.3 software. The bacterial population was selected on the basis of GFP expression; DsRED fluorescence intensity was analysed across the bacterial population, and the geometric mean of DsRED fluorescence was used to calculate the dilution of fluorescence (Helaine *et al.*, 2010).

#### Statistical analysis.

All data shown are either the mean and sd of a minimum of three independent experiments, or data from a single, representative experiment, reproduced on a minimum of three independent occasions. Statistical significance was calculated using Student's *t* test, where *P*<0.05 was judged to be significant.

## RESULTS AND DISCUSSION

### Visualization of lysosomes in macrophages

**Fig. 1.**
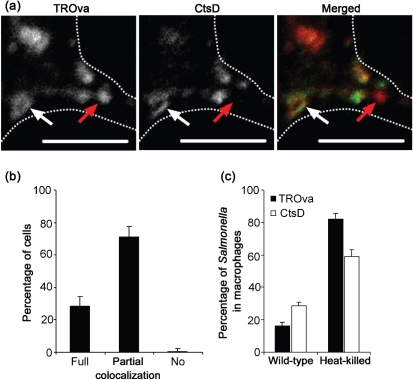
Identification of lysosomes in RAW264.7 macrophages. (a) Confocal microscopy images of a RAW264.7 macrophage pulse–chased with TROva (red in merged image) and labelled with anti-CtsD antibodies (green in merged image). The dotted white line indicates the cell membrane, white arrows indicate co-localization and red arrows indicate the absence of co-localization; bars, 5 µm. (b) Percentage of RAW264.7 macrophages containing full, partial or no co-localization between TROva and CtsD labelling. Data shown are the mean of three independent experiments in which a minimum of 50 cells were scored; error bars, sd. (c) Percentage of viable and heat-killed *S.* Typhimurium LT2 co-localized with TROva or anti-CtsD labelling in RAW264.7 macrophages 2 h after uptake. Data shown are the mean of at least three independent experiments; error bars, sd.

We first compared two methods for detecting lysosomes by light microscopy. In one, a fluid-phase marker, TROva, was pulse–chased into lysosomes. Cells were then fixed and labelled for the lysosomal enzyme CtsD (Garvis *et al.*, 2001). Analysis by confocal microscopy showed that both labels had a vesicular distribution (Fig. 1a). Almost all cells (99 %) contained at least partial pixel-to-pixel co-localization in the labelling patterns produced by TROva and CtsD (Fig. 1b). Although full co-localization was restricted to 28 % of cells, 71 % contained partial overlap, i.e. dual-labelled TROva- and CtsD-positive compartments (Fig. 1a, white arrow), as well as compartments distinctly labelled for one or the other marker (Fig. 1a, red arrow).

TROva is taken up from the extracellular environment and traffics through early and late endosomes before reaching lysosomes, whereas CtsD is delivered by the M6PRs to late endosomes from the trans-Golgi network (Ghosh *et al.*, 2003). Acidification triggers the release of the pro-enzyme from the receptor, whereupon cleavage generates the mature, hydrolytic protein found in the lysosomal lumen. One explanation for the lack of complete co-localization between these two markers is the ability of the anti-CtsD antibody to bind the immature, pro-form of the enzyme (data not shown). This would lead to the identification of pre-lysosomal compartments in addition to lysosomes containing the mature version of CtsD. Furthermore, lysosomes undergo fusion and kiss and run events with autophagosomes (Jahreiss *et al.*, 2008) and late endosomes (Mullock *et al.*, 1998, 2000; Pryor *et al.*, 2000). Differences in the sorting of TROva and CtsD at this stage might also give rise to the distinct patterns of labelling observed.

We next determined the extent of co-localization of these markers with positive and negative controls for avoidance of phagolysosomal fusion: viable or heat-killed *S*. Typhimurium strain LT2 in RAW264.7 macrophages fixed 2 h after uptake. The majority of viable bacteria avoided co-localization with either marker, with 28 % positive for CtsD compared with the 16 % positive for TROva (Fig. 1c). In contrast, 59 % and 82 % of heat-killed bacteria were positive for CtsD labelling and TROva, respectively (Fig. 1c). In view of the better discrimination between negative and positive controls obtained with TROva, this probe was used in subsequent experiments to assess levels of fusion between SCVs and lysosomes.

**Fig. 2.**
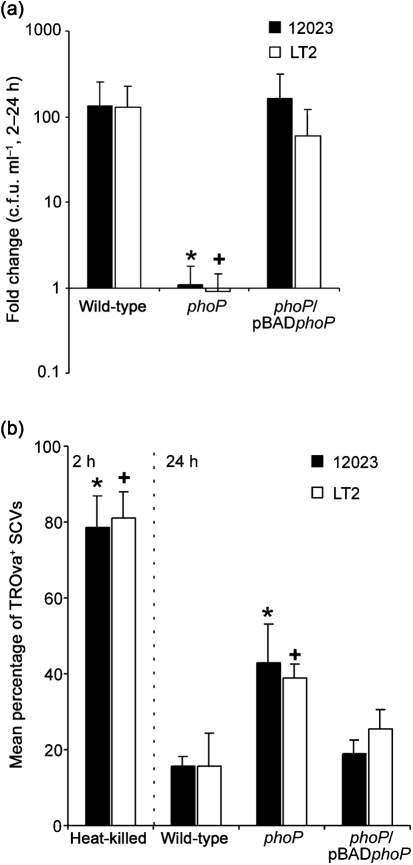
*S.* Typhimurium LT2 *phoP* mutant phenotype. (a) RAW264.7 macrophages were infected with 12023 or LT2 wild-type, *phoP* or *phoP*/pBAD*phoP* mutant *S.* Typhimurium in the presence of 0.2 % l-arabinose. Net growth between 2 and 24 h was calculated from the fold change in c.f.u. ml^−1^ recovered at these time points. (b) TROva-labelled RAW264.7 macrophages were infected with wild-type, *phoP* or *phoP*/pBAD*phoP* mutant *S.* Typhimurium in the presence of 0.2 % l-arabinose for 24 h. A positive control containing heat-killed wild-type *S.* Typhimurium was analysed 2 h post-uptake. The mean percentage of bacteria (as identified by antibody labelling) per cell that co-localized with TROva was scored by confocal microscopy. Data shown in (a) and (b) are the mean of three independent experiments (error bars, sd), and significant differences from the wild-type are indicated by an asterisk (12023) or cross (LT2), where *P*<0.05.

Due to the availability of the entire genome sequence of the *S*. Typhimurium LT2 strain at the time this study was initiated, it was decided to use this strain to construct the *pag* mutant strains. To validate the use of the LT2 strain, we first constructed a *phoP* mutant in this strain. The phenotype of the LT2 *phoP* mutant was indistinguishable from that of the 12023 *phoP* mutant used previously (Garvis *et al.*, 2001) with respect to intramacrophage growth and co-localization with TROva (Fig. 2a, b). Growth of both 12023 and the LT2 *phoP* mutant strain was restored, and the percentage of mutant bacteria that co-localized with TROva reduced from approximately 40 to 20 %, by the expression of *phoP* from pBAD*phoP* (Fig. 2a, b), showing plasmid-based complementation.

### A *pag* mutant strain partially reproduces the phenotype of the *phoP* mutant strain

**Fig. 3.**
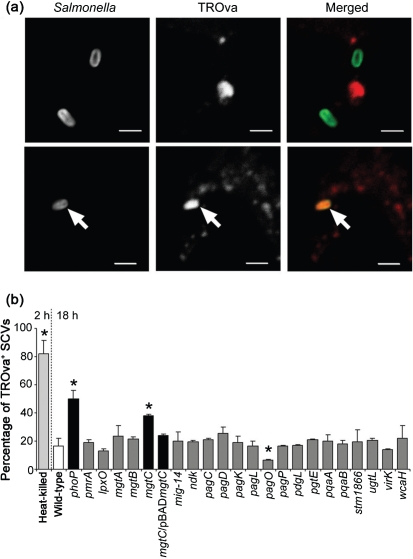
Analysis of TROva co-localization with *pag*-mutant *S.* Typhimurium strains in macrophages. (a) Characteristic images of *S.* Typhimurium (green in merged images) negative for TROva (red in merged images) co-localization (top panel) and heat-killed *S.* Typhimurium positive for co-localization (bottom panel) in RAW264.7 macrophages. Bars, 2 µm; arrow indicates TROva^+^ bacterium. (b) Mean percentage of *S.* Typhimurium per cell co-localized with TROva in RAW264.7 macrophages infected with the indicated bacterial strains for 2 h (heat-killed) or 18 h (all other strains). Data shown are the mean of at least three independent experiments (error bars, sd), and asterisks indicate a significant difference from the wild-type, where *P*<0.05.

Using the LT2 strain, mutations were made in *pmrA*, genes involved in modification of LPS, magnesium homeostasis, antimicrobial peptide (AMP) resistance, and genes with less well-characterized functions. TROva-labelled RAW264.7 macrophages were infected with wild-type or mutant bacteria for 18 h, or heat-killed bacteria for 2 h. Characteristic images of viable bacteria avoiding phagolysosomal fusion, and a non-viable bacterium exhibiting full co-localization, are shown in Fig. 3(a). For almost all the mutant strains analysed, the percentage of bacteria co-localizing with TROva was similar to that obtained for wild-type bacteria (Fig. 3b). The *pagO* mutant strain had a significantly lower percentage of co-localization compared with the wild-type strain. However, since the purpose of the screen was to identify mutants that could account for the *phoP* mutant phenotype, this was not studied further. Only the *mgtC* mutant strain exhibited a significantly higher mean percentage of co-localization: 38 % of bacteria per cell were positive for phagolysosomal fusion, compared with 50 % for the *phoP* mutant and 15 % for the wild-type strain. This phenotype was rescued by the expression of *mgtC* from pBAD*mgtC* (Fig. 3b).

*mgtC* encodes a 23 kDa protein required for *S*. Typhimurium growth within macrophages (Blanc-Potard & Groisman, 1997; Rang *et al.*, 2007). In addition, an *mgtC* mutant strain has also been found to be deficient for growth in magnesium-deprived conditions *in vitro* (Blanc-Potard & Groisman, 1997). *mgtC* is in an operon that includes the magnesium transporter-encoding gene *mgtB* (Hmiel *et al.*, 1989), and although there is no evidence that supports a direct role for MgtC as an ion transporter (Günzel *et al.*, 2006; Moncrief & Maguire, 1998), the putative inner membrane location of MgtC (Rang *et al.*, 2007), the importance of this protein for *in vitro* growth, and the partial rescue of intramacrophage growth of *mgtC* mutant bacteria upon addition of MgCl_2_ (Blanc-Potard & Groisman, 1997), all suggest that MgtC promotes growth within environments limited in metal ions. It is therefore unclear how it might enable avoidance of phagolysosomal fusion. In view of this result, we reappraised the apparent contribution of PhoP/Q to survival and replication in macrophages (Garvis *et al.*, 2001).

### Contribution of PhoP to replication in macrophages

**Fig. 4.**
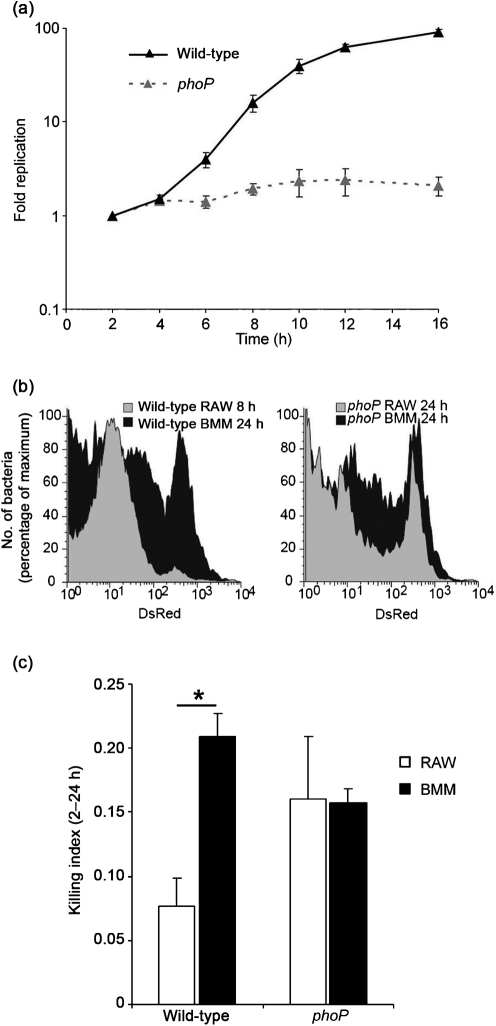
PhoP/Q is required for *S.* Typhimurium replication in macrophages. (a) Replication, quantified from the fold change in fluorescence, of wild-type and *phoP* mutant pDiGc *S.* Typhimurium in RAW264.7 macrophages. Data shown are the mean of three independent experiments; error bars, sd. (b) Representative histograms of the fluorescence intensity of wild-type (left) or *phoP* mutant (right) pDiGc *S.* Typhimurium recovered from RAW264.7 macrophages and BMM at 8 and 24 h, as indicated. (c) Killing indices (generations h^−1^) of wild-type and *phoP* mutant pDiGc *S.* Typhimurium in RAW264.7 cells and BMM between 2 and 24 h. Data shown are the mean of three independent experiments (error bars, sd), and asterisks indicate a significant difference between samples, where *P*<0.05.

As LT2 and 12023 *S*. Typhimurium strains displayed similar phenotypes in terms of lysosomal co-localization and propagation in macrophages (Fig. 2), and since the kinetics of replication, killing and overall growth of the 12023 strain had already been thoroughly characterized in macrophages (Helaine *et al.*, 2010), we assessed the contribution of PhoP/Q to replication using strain 12023. RAW264.7 macrophages were infected with wild-type or *phoP* mutant strains carrying pDiGc. This plasmid encodes constitutive GFP as a marker for bacterial cells, and arabinose-inducible DsRED, the preformed pool of which is diluted between daughter cells with each bacterial division upon removal of the inducer. Replication is quantified according to decreases in DsRED fluorescence intensity (Helaine *et al.*, 2010). Dilution of DsRED fluorescence in *S*. Typhimurium recovered from RAW264.7 macrophages was monitored by flow cytometry over a 16 h period and bacterial replication calculated from the fold change in geometric mean fluorescence. This revealed an approximately 100-fold increase in wild-type bacteria between 2 and 16 h (Fig. 4a), which corresponded to a mean rate of 0.46 generations h^−1^. In contrast, the phenotype of *phoP* mutant bacteria became apparent 4 h post-uptake, with the bacteria exhibiting almost no replication (Fig. 4a) and a mean of approximately one cell division over the full course of the experiment.

Single-cell analysis by flow cytometry of bacteria recovered from RAW264.7 macrophages showed that wild-type *S.* Typhimurium replicated in a relatively synchronous wave, as evidenced by the high frequency of events within a narrow range of fluorescence intensities. This is shown for bacteria recovered after 8 h of infection (Fig. 4b, left panel, grey profile), but was consistently reproduced at each time point analysed (data not shown and Helaine *et al.*, 2010). In contrast, wild-type bacteria recovered from BMM after 24 h exhibited a broader, more heterogeneous distribution of fluorescence intensities and therefore replicative capability (Fig. 4b, left panel, black profile). Presumably this reflects the increased stress to which bacteria were subjected in BMM compared with RAW264.7 macrophages, and was accompanied by only a 10-fold increase in bacteria in these cells (data not shown and Helaine *et al.*, 2010).

The distribution of fluorescence intensities of *phoP* mutant bacteria in RAW264.7 macrophages or BMM over a 24 h period was similar, revealing a heterogeneous distribution of fluorescence intensity, and therefore replicative ability, across the bacterial population (Fig. 4b, right panel). The extent of replication of *phoP* mutant bacteria in BMM macrophages was similar to that in RAW264.7 cells (data not shown). The profiles of *phoP* mutant bacteria in BMM and RAW264.7 macrophages (Fig. 4b, right panel) more closely resembled those of wild-type bacteria in BMM rather than RAW264.7 macrophages (Fig. 4b, left panel), indicating that the *phoP* mutant strain sustains a similar degree of stress, with consequent effects upon replication, irrespective of macrophage type.

We then analysed whether this effect upon the replicative potential of *phoP* mutant bacteria was accompanied by an increased susceptibility to killing. Differences between the rates of replication and net growth for wild-type and *phoP* mutant strains were used to calculate killing indices (Helaine *et al.*, 2010). Relatively little bacterial loss was observed for wild-type *S*. Typhimurium recovered from RAW264.7 macrophages between 2 and 24 h, generating a killing index of 0.08 generations h^−1^ (Fig. 4c). This almost tripled to 0.21 generations h^−1^ for bacteria recovered from BMM (Fig. 4c; Helaine *et al.*, 2010), confirming that these macrophages have a greater bactericidal capacity. Despite this difference in killing activity, there was no difference in the relatively high killing indices obtained for *phoP* mutant bacteria in the different macrophages, representing a loss of 0.16 generations h^−1^ between 2 and 24 h (Fig. 4c). This shows that bacteria lacking PhoP are highly sensitive to both RAW264.7 macrophages and BMM, and that the increased bactericidal capacity of BMM has no greater effect on this mutant strain. Therefore, the factors to which the PhoP/Q regulon enables intracellular adaptation are likely to be a common feature of RAW264.7 macrophages and BMM.

### Kinetics of avoidance of phagolysosomal fusion

**Fig. 5.**
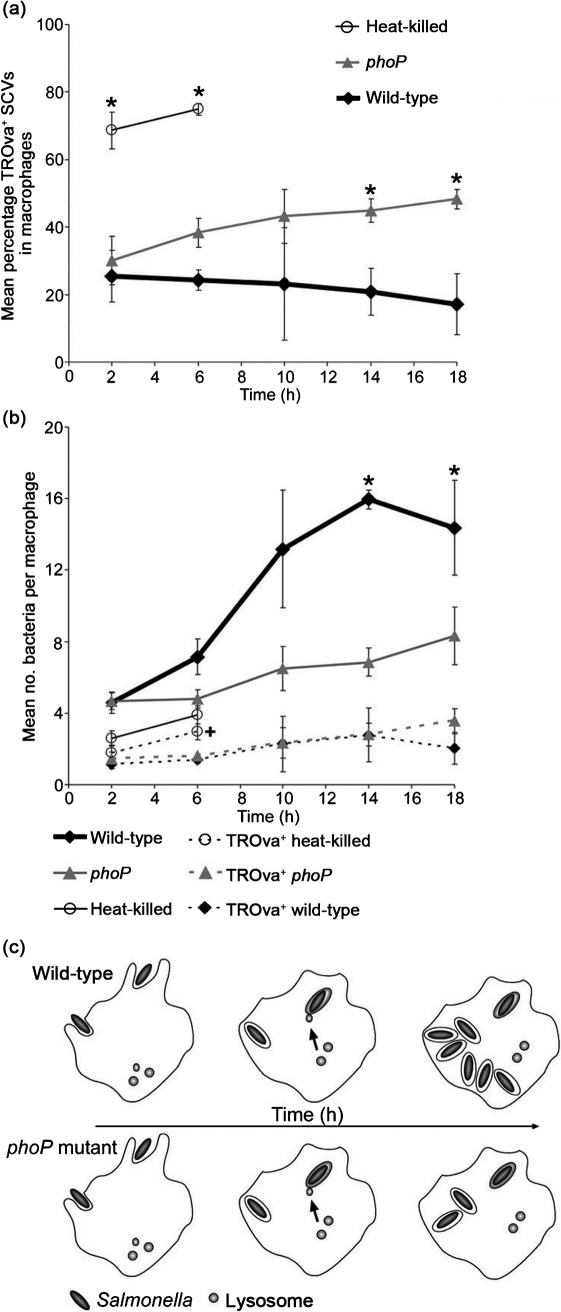
Kinetics of SCV–lysosome interactions in RAW264.7 macrophages. (a) Mean percentage of bacteria per cell co-localized with TROva in RAW264.7 macrophages infected with wild-type, *phoP* mutant or heat-killed *S.* Typhimurium at the times indicated. Data shown are the means of at least three independent experiments (error bars, sd), and asterisks indicate a significant difference from the wild-type at the corresponding time point, where *P*<0.05. (b) Mean total number of bacteria per cell and mean number of TROva^+^ bacteria per cell in RAW264.7 macrophages infected with wild-type, *phoP* mutant or heat-killed *S.* Typhimurium. Data shown are the mean of at least three independent experiments (error bars, sd), and asterisks indicate a significant difference between wild-type and *phoP* mutant bacteria, while a cross indicates a significant difference between wild-type and heat-killed bacteria, where *P*<0.05. (c) Illustration of how differences in growth rates between wild-type and mutant bacterial strains affect the interpretation of the avoidance of phagolysosomal fusion on a proportional basis. In each case only one phagosome fuses with lysosomes, but the overall growth defect of the mutant results in a higher proportion of phagosomes that have undergone fusion.

As the *phoP* mutant strain appeared to be equally stressed in RAW264.7 macrophages and BMM, despite the increased bactericidal activity of the latter, we re-examined the kinetics of phagolysosomal fusion in RAW264.7 macrophages at early and late time points for both wild-type and *phoP* mutant *S*. Typhimurium. Macrophages were pulse–chased with TROva and infected with opsonized bacteria. Between 25 and 17 % of wild-type bacteria per cell co-localized with TROva at selected time points throughout an 18 h infection (Fig. 5a). Although the percentage of *phoP* mutant bacteria co-localized with TROva increased with time, reaching 45 and 48 % of bacteria by 14 and 18 h post-inoculation, respectively, in broad agreement with previous work from our laboratory (Garvis *et al.*, 2001), the percentage of co-localization at 2 h was very similar to that of the wild-type strain (Fig. 5a). In contrast, almost 70 % of heat-killed bacteria were positive for TROva labelling at 2 and 6 h post-inoculation, indicating rapid phagolysosome maturation.

To take account of the observed difference in the replication rates of the wild-type and *phoP* mutant strains (Fig. 4a) in the analysis of phagolysosomal fusion, the total numbers of bacteria per macrophage, and those co-localized with TROva, were compared by microscopy (Fig. 5b). A significant difference between wild-type and *phoP* mutant bacteria was apparent in the total number of bacteria per cell: from 6 h onwards an increase in the number of wild-type bacteria per cell was observed, which reached a mean bacterial load of 14–16 bacteria per cell by 14 and 18 h (Fig. 5b); at these time points, macrophages infected with *phoP* mutant *S*. Typhimurium only contained seven to eight bacteria. This difference in the intracellular numbers of wild-type and *phoP* mutant strains is also in agreement with their divergent kinetics of replication measured by fluorescence dilution (Fig. 4a).

Whether infected with wild-type or *phoP* mutant *S*. Typhimurium, macrophages contained a mean of four to five bacteria at 2 h post-uptake. In each case, one to two of these bacteria had co-localized with TROva (Fig. 5b). As the infection proceeded, the number of TROva-positive bacteria only increased by approximately one per cell, and at no point was there a significant difference in the number of wild-type or *phoP* mutant *S*. Typhimurium positive for TROva labelling (Fig. 5b). Therefore, we conclude that the PhoP/Q regulon does not directly affect SCV–lysosome interactions, but does enable proliferation of the non-lysosomal population of bacteria. As a result, the few *phoP* mutant bacteria undergoing phagolysosomal fusion constitute a greater proportion of the total intracellular population than do phagolysosomal wild-type bacteria, consistent with previous results (Garvis *et al.*, 2001). These interpretations are summarized in Fig. 5(c), and highlight how differences in the intracellular growth of bacterial strains can generate misleading conclusions when data are expressed as a proportion of the population rather than in absolute terms.

Although there was no detectable difference between wild-type and *phoP* mutant bacteria, the number of heat-killed bacteria that co-localized with TROva was significantly higher than that of the wild-type at 6 h post-uptake. This indicates that there are factors other than the PhoP/Q regulon that enable *S.* Typhimurium to escape phagolysosomal fusion. Although the SPI-2 T3SS has been implicated in this phenomenon (Uchiya *et al.*, 1999), a recent study by our group indicates that the SPI-2 T3SS is not involved in resistance to macrophage killing mechanisms (Helaine *et al.*, 2010), and future work is required to establish the identity of the relevant bacterial molecules.

Genes of the PhoP/Q regulon are involved in several functions, including magnesium transport (Blanc-Potard & Groisman, 1997; Soncini *et al.*, 1996). AMPs, produced by a range of host cells including macrophages, have activity against *S*. Typhimurium (Beuzón *et al.*, 2002; Blanc-Potard & Groisman, 1997; Hiemstra *et al.*, 1993, 1999; Soncini *et al.*, 1996). *In vitro* studies indicate that some PhoP-activated genes (*pag*s), including *mig-14*, *virK* and *pagP*, increase resistance to AMPs such as polymyxin B and protegrin-1 (Brodsky *et al.*, 2002; Detweiler *et al.*, 2003; Guo *et al.*, 1998); the partial rescue of growth of a *phoP* mutant strain in BMM lacking the AMP CRAMP suggests that this also occurs in the intramacrophage environment (Rosenberger *et al.*, 2004). Components of the PhoP/Q regulon have been shown to be involved in the tolerance response to inorganic acid stress at pH 4.5 (Bearson *et al.*, 1998). The pH of the SCV drops rapidly to between pH 4 and 5 (Rathman *et al.*, 1996). Therefore, an acid tolerance response is likely to be important for *S*. Typhimurium to adapt to this environment and efficiently express other factors required for survival and replication within the phagosomal environment. Resistance to AMPs and resistance to acidic pH are therefore likely to be important responses enabling PhoP/Q-dependent replication of *Salmonella* in macrophages and virulence in its hosts.
